# Supplementary material for: Precisely timed inhibition facilitates action potential firing for spatial coding in the auditory brainstem
Source: Nat Commun. 2018 May 2;9:1771. doi: 10.1038/s41467-018-04210-y (PMC5932051; doi:10.1038/s41467-018-04210-y)
Supplement: Supplementary file 1 — Supplementary Information [file 41467_2018_4210_MOESM1_ESM.pdf]

Supplementary information

Precisely timed inhibition facilitates action potential firing for spatial coding in the auditory brainstem

Beiderbeck et al.

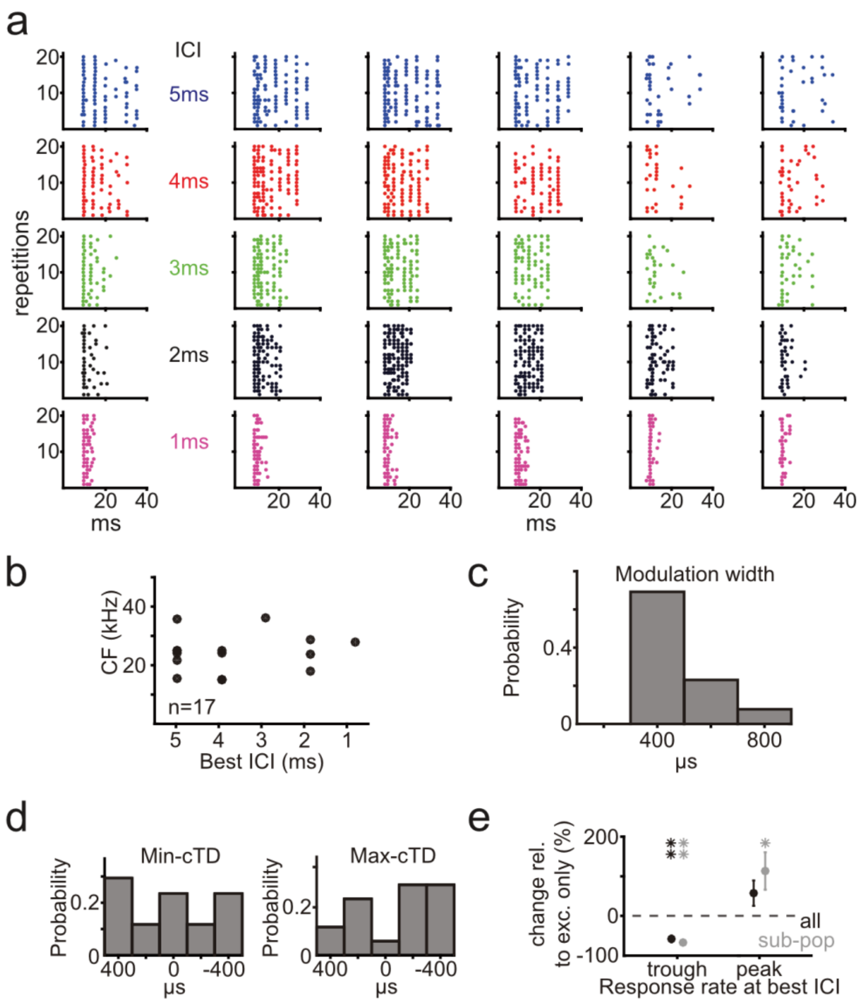

Supplementary Figure 1. Quantifications of sensitivity of LSO neurons to ICI and ITD.

(a) Dot-raster displays from an example neuron (CF: 24.3 kHz) during (ipsilateral) excitatory only stimulation (left panel, 74 dB SPL, 20 repetitions) with click-trains at various ICIs (5-1 ms, color-coded) and during binaural stimulation (right panel, ipsi: 74 dB SPL, contra: 79 dB SPL, 20 repetitions) with click-trains at various ICIs (5-1 ms, color-coded) and and various ITDs (400 to -400 µs, stepsize: 200 µs). (b) Best ICIs of individual neurons did not depend on CF, but were biased towards larger ICIs, as 13/17 neurons had best ICIs of 5 ms or 4 ms. (c) Response rates were steeply modulated by ITD, as the median width in ITD between the min- and max-ITD was only 400 µs. (d) Both min-ITDs and max-ITDs varied across the population. (e) Changes in binaural response rates at best ICIs relative to average monaural mean response rates: Average mean response rates at min-ITD are shown for all neurons (black, n=17) and for a subpopulation of neurons whose absolute response rates at the min-ITDs fell below their monaural mean response rate (grey, n=15). Min-rates were significantly lower than mean rates for excitatory only (-57.8 % ± 8.3 %, p=0.000003, Student's t-test, and -66.9 % ± 6.1 %, p=0.000003, Student's t-test, respectively). Mean rates at max-ITDs exhibited a sizable increase of binaural response rates in the entire population (black, 57.6 % ± 31.9 %, p=0.09, Student's t-test) and a significant increase of response rates in a subpopulation of neurons whose mean responses at max-

ITD superseded the response rate for exc. only stimulation (grey, n=10, 113.5 % ± 47.1 %, p=0.04, Student's t-test). Data points are mean ± s.e.m.

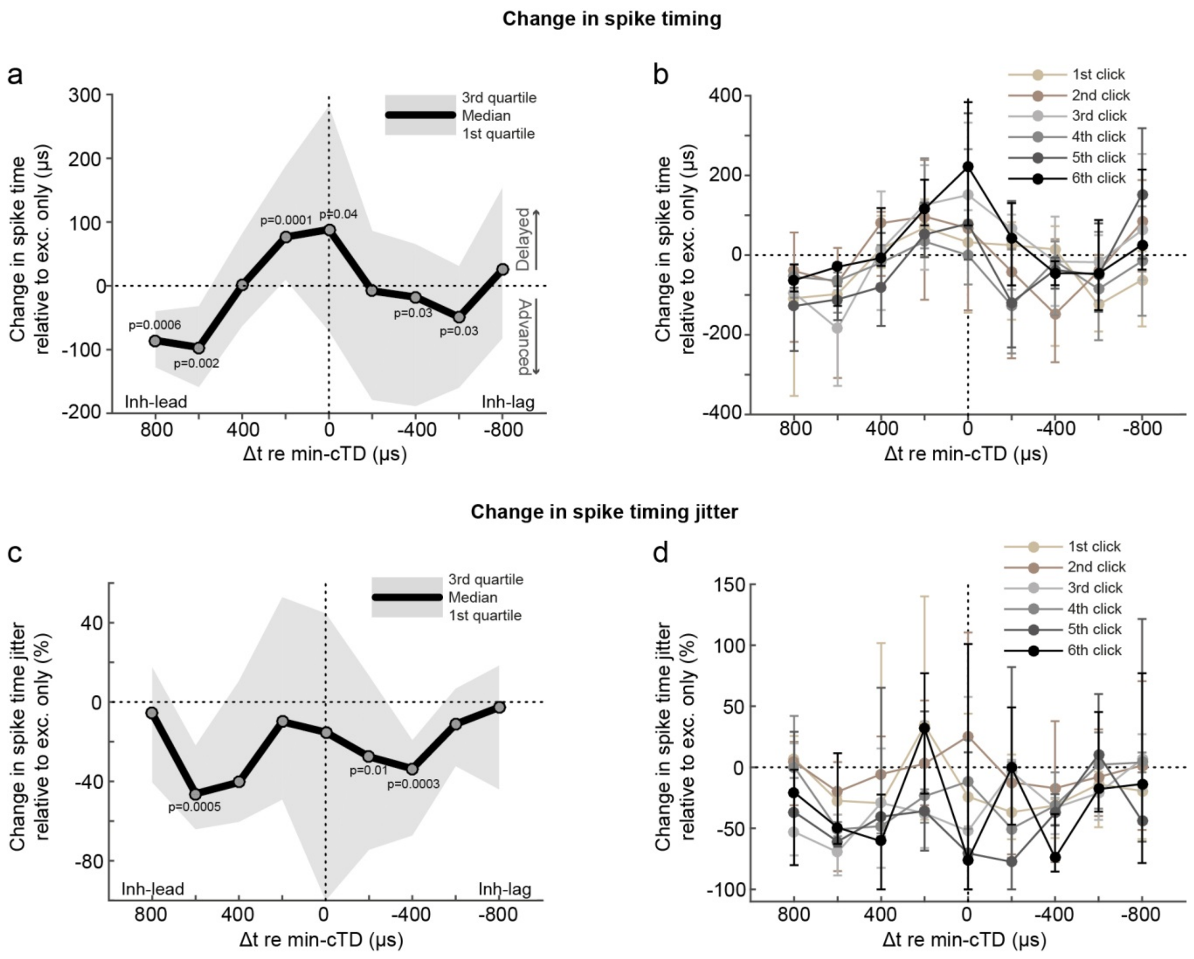

Supplementary Figure 2. Precisely times inhibition modulates spike timing and reduces jitter. Changes in spike timing and jitter were observed as a function of ∆t (n=15 neurons from 11 animals). (a) Compared to the timing during excitation only, spikes occurred significantly earlier if inhibition led excitation by 600 µs (median: 96.6 µs, p=0.002; Wilcoxon signed rank test;) to 800 µs (median: 85.7 µs, p=0.0006; Wilcoxon signed rank test;). Interestingly, the lead of inhibition by 600 µs coincided with the delay of highest spike facilitation (Fig. 3c). Spikes were also advanced significantly, but more modestly, if inhibition lagged excitation by 400 µs or 600 µs (medians of 17.7 µs and 49.1 µs, p=0.03 and p=0.03, respectively; Wilcoxon signed rank test;). Conversely, if inhibition occurred simultaneously (∆t = 0 µs) or led excitation by 200 µs, spike times were significantly delayed compared to monaural stimulation (88.3 µs and 76.6 µs, p=0.04 and p=0.0001, respectively; Wilcoxon signed rank test). (b) The tendency of bidirectional spike timing shifts as a function of the input delay was similar for all clicks in the train. (c) Jitter was decreased on average across the Δts tested. Decreased jitter represents an increase in timing precision, which was highest for ∆t=600µs (inhibition leading, median: -46.2%, p=0.0005): Significant jitter reduction was further reached for ∆t=-200µs and ∆t=-400µs (inhibition lagging, median: -27.6% and -33.6%; p=0.01 and p=0.0003, respectively, paired Wilcoxon signed rank test). (d) Changes in spike timing jitter for individual clicks in the train. Jitter reduction tended to be higher for clicks later in the train, particular when inhibition was leading. Conventions as in Fig. 3.

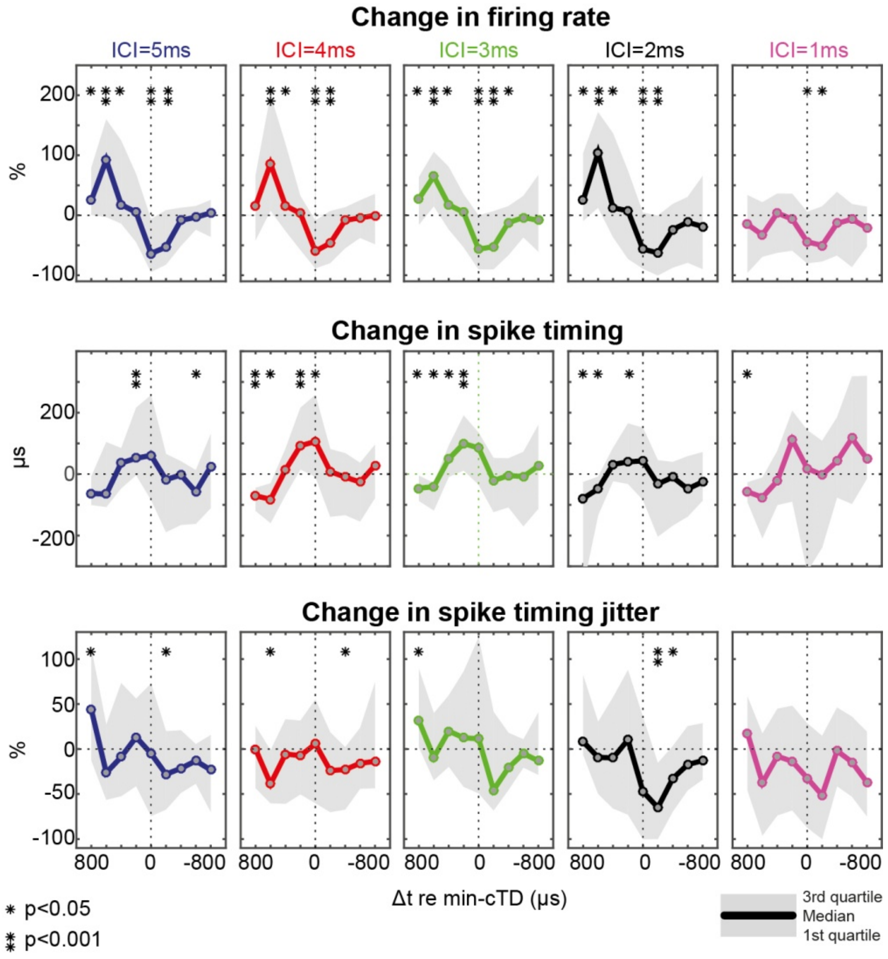

Supplementary Figure 3. ITD-dependent modulation of rate, timing and jitter across ICIs.
Effects of precisely timed inhibition are present at all ICIs across the population of neurons (n=17 for spike rate, n=15 for spike timing and jitter). Average changes (median ± 25th and 75th quantile) to the entire click train compared to median responses during exc. only at all ICIs (from left to right: 5 ms to 1 ms, color-coded) are plotted. Significant changes are denoted by asterisk based on the paired Wilcoxon signed rank test.

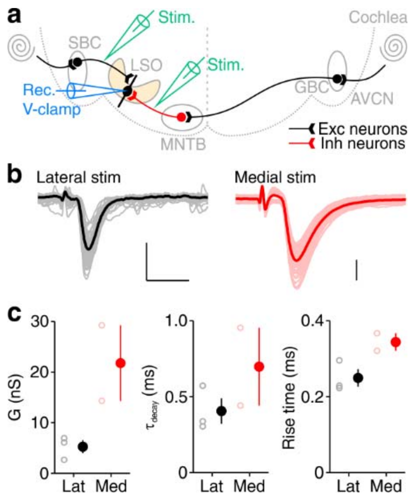

Supplementary Figure 4. Acquisition of synaptic waveform conductance templates. (a) Schematic illustrates the recording and stimulation paradigm. (b) Example traces from stimulation lateral (left) and medial (right) to the recorded cell to elicit excitatory and inhibitory currents, respectively. Light traces are individual trials, and bold traces are the average of 50 traces. Scale bars: 200 pA, 2 ms. (c) Conductance (left), decay tau (middle) and rise time (right) are shown for individual experiments (light markers) and averages (filled markers) of lateral and medial stimulation.

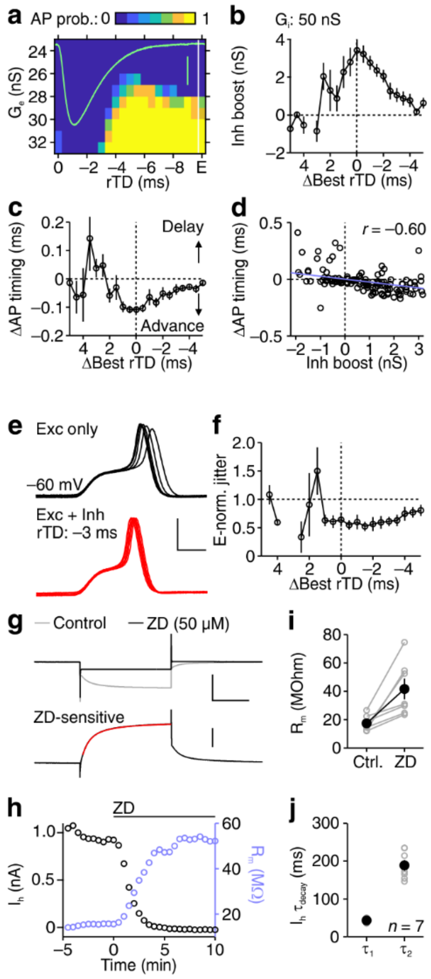

Supplementary Figure 5. Effects of inhibitory boost on spike timing and characterization of I_h currents. (a) AP probability heat map as a function of rTD and G_e, with an IPSP trace overlaid. Red lines indicate the interpolated G_e value causing 50% AP probability. "E": excitation only, G_i: 50 nS. (c) Peak-aligned (Best rTD) before averaging (Supplementary Fig. 4e), where zero represents the average Best rTD across the whole sample. (b) Population average AP conductance thresholds of inhibition relative to excitation alone plotted against relative Best rTD quantifies the timing-dependent inhibitory boost (Inh boost). (c) Average AP onset timing with inhibition relative to excitation alone plotted against relative Best rTD reveals timing conditions that advance and delay APs. (d) Individual trials of AP timing shifts (c) plotted against inhibitory boost (b) shows a small, but significant linear correlation ($P = 2.06 \times 10^{-18}$). (e) Overlaid traces from an example recording of excitation only (top, s.d. = 68 μS) and excitation with an inhibition (bottom, rTD = –3 ms, s.d. = 28 μs) show tightening of spike timing enforced by inhibition. Scale bar: 50 mV, 0.5 ms. (f) Average jitter, normalized to excitation alone (E-norm) for the population of recordings show a generalized reduction in spike timing jitter that is substantial particularly around the Best rTD ($P = 7.38 \times 10^{-7}$, one-sample t-Test). (g) Top: example current traces for a 500 ms voltage-clamp step protocol from –50 to –80 mV before (Control, gray) and after (black) ZD wash-in. Bottom: the ZD-sensitive current, calculated by subtracting the control currents from the those in ZD. Red line indicates a double-exponential fit. (h) Time course of the example experiment in (g), showing the complete abolishment of I_h (black) as well as an increase

in input resistance (blue). (i) Input resistance before (17.4 ± 2.0 MΩ) and after (41.5 ± 7.3 MΩ) ZD wash-in (n = 7 cells, P = 0.01, paired t-Test). (j) First (τ_1, 43.1 ± 2.1 ms) and second (τ_2, 188.9 ± 14.2 ms) decay time constants from fitted Ih currents.

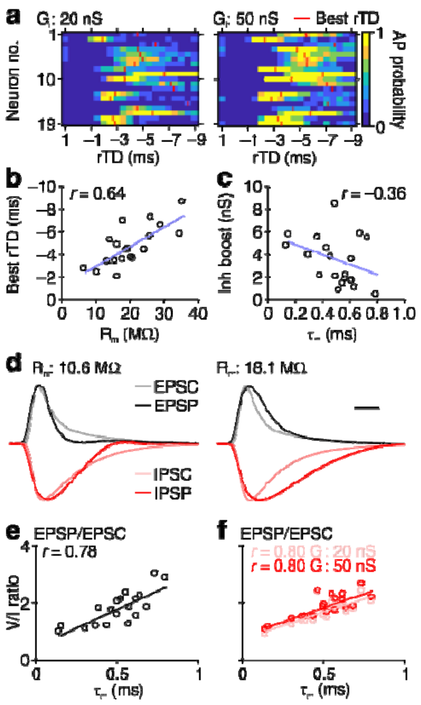

Supplementary Figure 6. Kinetic determinants of best rTD and inhibitory boost. (a) Heat maps of PIF functions with G_i = 20 nS (left) and 50 nS (right) for all 19 recordings of single-event stimulation (Fig. 4a,b and Supplementary Fig. 5a–f) . Red lines indicate the cell-wise Best rTDs. (b) Plot of Best rTD against input resistance shows a linear correlation (P = 0.003). (c) Plot of inhibitory boost (at Best rTD) against membrane time constant shows trend of faster membrane kinetics to express more inhibitory boost (P = 0.113). (d) Overlaid traces of normalised EPSGs (gray) and EPSPs (black), as well as IPSGs (pink) and IPSPs (red) for example recordings of relatively low input resistance (left) and higher input resistance (right) illustrate how membrane kinetics can strongly influence the kinetic transformation from synaptic conductance to membrane potential. Scale bar: 0.5 ms. (e,f) Plots of the ratio between PSP to PSG half-width for excitatory (e, $P = 7.10 \times 10^{-5}$) and inhibitory (f, G_i = 20 nS, $P = 4.01 \times 10^{-5}$; G_i = 50 nS, $P = 3.73 \times 10^{-5}$) events.
